# Children, care, career – a cross-sectional study on the risk of burnout among German hospital physicians at different career stages

**DOI:** 10.1186/s12995-014-0041-6

**Published:** 2014-12-03

**Authors:** Astrid Richter, Petya Kostova, Volker Harth, Ralf Wegner

**Affiliations:** Institute for Occupational and Maritime Medicine (ZfAM), University Medical Center Hamburg-Eppendorf, Seewartenstraße 10, Haus 1, 20459 Hamburg, Germany

**Keywords:** Burnout, Gender, Hospital physicians, Leadership, Work-family conflict

## Abstract

**Background:**

With the increasing number of female medical students physicians’ need for work-life balanced hospital jobs rises at all career stages. The Working Time Act (Arbeitszeitgesetz, ArbZG), an implementation of the European Working Time Directive into German law in 2004, should have improved the general conditions for creating flexible work. Nevertheless, the vast majority of female physicians still report an incompatibility of work and family. So far, little is known about mothers working on leading positions in the medical field. The presented study focuses on gender differences in the level of emotional exhaustion between child-rearing junior and senior physicians and different predictors of burnout.

**Methods:**

Three years after the ArbZT-enactment, 994 physicians from the listed hospital physicians in the Medical Register of the city of Hamburg participated in the cross-sectional study and completed a 60-item questionnaire (return rate of 46,5%). The questionnaire included a 22-item version of the German translation of the Maslach Burnout Inventory whereat emotional exhaustion was interpreted as the crucial predictor of burnout. Results of an univariate covariance analysis and regression analyses are reported.

**Results:**

In the level of emotional exhaustion no gender differences were found between junior and senior physicians with children in the overall analysis. Support by the superior was the only overall predictor of burnout. Female senior physicians having children presented the highest risk of burnout. Only in this group parenting contributed significantly to the risk of burnout.

**Conclusions:**

Support by the superior and the relationship to colleagues are generally important predictors of burnout among hospital physicians. Parenthood only gets a crucial influence on psychomental health for female senior physicians. Still conservative role models are common in this group, thus dealing with the triple burden of work, leadership responsibility and child rearing seems to be a special female challenge. Innovative approaches of human resource policy need to be implemented to improve the reconciliation of family and working life.

## Abstract (Deutsch)

**Hintergrund:**

Mit der zunehmenden Zahl von Medizinstudentinnen wächst in den Krankenhäusern auf allen Karrierestufen auch das Bedürfnis nach Arbeitsstellen, die eine Balance zwischen Beruf und Privatleben ermöglichen. Im Jahr 2004 wurde in Deutschland mit dem Arbeitszeitgesetz (ArbZG) die europäische Arbeitszeitrichtlinie umgesetzt, welche die Arbeitszeitgestaltung flexibilisieren sollte. Doch auch heute empfindet ein Großteil der Ärztinnen, dass Beruf und Familie unvereinbar sind. Bis jetzt ist wenig über jene berufstätigen Mütter bekannt, die als Führungskräfte im medizinischen Bereich arbeiten. Die vorliegende Studie untersucht Geschlechtsunterschiede in der wahrgenommenen emotionalen Erschöpfung zwischen Eltern, die als Assistenzärzt(inn)en und Ober- bzw. Chefärzt(inn)en arbeiten, sowie verschiedene Prädiktoren von Burnout.

**Methoden:**

Vier Jahre nach Umsetzung des Arbeitszeitgesetzes nahmen 994 Ärzt(inn)en aus dem Ärzteregister der Stadt Hamburg an der Querschnittsstudie teil. Sie füllten einen Fragebogen mit 60 Items aus, was einer Rücklaufquote von 46,5% entspricht.

Der Fragebogen enthielt eine deutsche Übersetzung des Maslach-Burnout-Inventars mit 22 Items, wobei die emotionale Erschöpfung als der entscheidende Prädiktor für Burnout angesehen wurde. Dargestellt werden die Ergebnisse einer univariaten Kovarianzanalyse und von Regressionsanalysen.

**Ergebnisse:**

Bei der emotionalen Erschöpfung ergaben sich in der gruppenübergreifenden Analyse keine Geschlechtsunterschiede zwischen Eltern, die als Assistenzärztinnen und Assistenzärzte oder als Ober- bzw. Chefärztinnen und -ärzte arbeiten. Die wahrgenommene Unterstützung durch die Vorgesetzte bzw. den Vorgesetzten stellte den einzigen gruppenübergreifenden Prädiktor für Burnout dar. Ober- bzw. Chefärztinnen mit Kindern wiesen das höchste Burnoutrisiko auf. Nur in dieser Gruppe nahm der Umstand ihrer Elternschaft signifikanten Einfluss auf das Risiko, ein Burnout zu entwickeln.

**Schlussfolgerungen:**

Die wahrgenommene Unterstützung durch die Vorgesetzte bzw. den Vorgesetzten wie auch das Verhältnis zu den Kolleg(inn)en sind bedeutsame Prädiktoren für Burnout bei Krankenhausärzt(inn)en. Ein Kind zu haben, wirkt sich erst in der Gruppe von Frauen mit Vollzeitanstellung und Führungsaufgabe bedeutsam und kritisch auf die psychomentale Gesundheit aus. In der genannten Gruppe dominieren auch heute noch konservative Rollenmodelle. Somit scheint der Umgang mit dieser Belastung in besonderem Maße eine Herausforderung für Frauen zu sein. In der Personalpolitik von Kliniken sind innovative Ansätze gefragt, um die Vereinbarkeit von Familie und Beruf zu gewährleisten.

## Background

During the last two decades a gradually paradigm shift in the medical profession has become apparent. According to OECD statistics medicine has become increasingly female in most European countries [1]. As data from Germany indicates, between 1991 and 2010 the proportion of women in the medical profession has raised from 33,6% to 43% [2]. Although, in the meantime, women make up the majority of medical students as well as junior physicians [3], they are still under-represented in medical leading positions [4,5]. In 2010 only 26% of Germany’s senior physicians and 9% of chief physicians were female [3].

Nevertheless, due to the demographic change and thus, an already internationally noticeable shortage of skilled workers in the medical field [6-8], women with children should assume more leading positions in the future [9]. In line with this development the question of reconciliation of family and employment becomes more urgent, especially for female senior physicians.

Hoff et al. [10] found out that traditional role models dominate partnerships with at least one person working as a physician. Therefore female physicians engage stronger in family and are more likely to have a full-time working spouse than their male colleagues do. Other studies [11,12] support this finding of women working “the second shift” [13] in household and child-rearing, which is even more likely in relationships of two physicians than between female physicians and non-medicals spouses. Frone et al. [14] stated that a lack of time to meet the demands in both domains of work and family can lead to conflicts, dividing these into family-work conflicts (FWC) and work-family conflicts (WFC). FWCs occur when family life interferes with work, whereas WFCs, when work interferes with family life. In fact, German physicians, particularly junior physicians, experience work-family conflicts independently of their sex more often than the general population [15]. Job stress, defined as the self-assessed level of job-caused exhaustion and inability to get cognitively distanced from the job at home [16], as well as the number of hours worked per week, inflexible work and shiftwork [17] are positively linked with WFCs. In addition, the longer someone works, the more probable WFCs and, as a possible consequence, burnout appear to be [16,18-20]. No gender differences were found for these connections in the studies cited.

According to Maslach et al. [21] burnout describes a ”prolonged response to chronic emotional and interpersonal stressors on the job”. The three dimensions emotional exhaustion, depersonalization and reduced personal accomplishment define this construct, whereof emotional exhaustion is the key aspect of burnout [21-24]. Emotional exhaustion includes the stress dimension of burnout as well as the impression of exhausted and depleted emotional resources. The second dimension, depersonalization, is characterized by social withdrawal and an impersonal, cynical response towards work and associated people such as colleagues and patients. The impression of reduced competence and success in work describes personal accomplishment as the third dimension of burnout [21,25]. Results for gender differences on burnout are inconsistent across diverse studies [26]. The features of hospital physicians’ work with a high work load, conflicts in dependency, responsibility and autonomy, along with long hours of duty and sleep deprivation, make role conflicts probable and can increase the risk of enduring stress and burnout [25,27,28]. A third of the physicians in a Swiss longitudinal study suffered from persistent intensive work-related stress [29]. Perceived stress and burnout are linked to each other as work-related stress can increase emotional exhaustion, which itself is the strongest predictor of burnout [30,31]. The point prevalence for European physicians developing burnout lies between 40 and 50 percent [31]. As we pointed out in a former study, the risk of burning out for hospital physicians has increased since the 1990s [32]. Nevertheless, the Working Time Act (Arbeitszeitgesetz, ArbZG), which implemented the European Working Time Directive into German law in 2004, aimed to improve the general conditions for creating flexible work. Flexible work time models as well as child care facilities matching work schedules and part-time jobs can be useful steps to reduce conflicts between work and family for employed parents [20,33]. Unfortunately, these measures are still rarely offered to hospital physicians in Germany. Taking this into account, with 88% the vast majority of German female physicians consider work and family to be currently incompatible [34].

As a consequence, potential qualified female employees with children are lost to the health care system temporarily or longer-term through opt out of hospital service or reduction on part-time jobs [33,35]. Female physicians who foster a career in hospitals often decide against having children at all or have less children than their male colleagues at the same career level [3,36]. This development can not only be observed in German but also in the United States [37].

In order to preserve the high level health-care in future, it is crucial to retain as many qualified physicians as possible in hospital employments. Due to the development of the recent student numbers in medicine, it is probable, that the group of female physicians with children who wish to work in full-time positions with career prospects will grow in the future. Therefore working conditions should respond to the specific requirements of the female medical staff, in order to make work and family life compatible at different career stages.

Despite the foreseeable development, knowledge about this group of physicians with the triple burden of work, leading position and child-rearing is still scarce. As far as we know, there exists no study focusing on the amount of emotional exhaustion of those physicians with children, especially women, trying to strike a balance between career and family life and to prevent from burning out. Purpose of the present study is to contribute to the knowledge to fill this gap, raising the following research questions:

1. Do male and female physicians rearing children differ in their perception of emotional exhaustion depending on their career stage? (research question 1)

One part of research literature rather speaks against gender differences on emotional exhaustion and work family conflicts for child rearing physicians (15,17-19). On the other hand, for employed parents it is stated that mothers, contrary to fathers, even have a lower burnout risk than the whole population [26]. Nevertheless, female managers report to carry the major burden of family problems compared to their spouses [38]. Besides taking leadership responsibility into account we expect differences on emotional exhaustion between the relevant groups.

Hypothesis: Among German hospital physicians with children there exist significant differences between the reported levels of emotional exhaustion at different career stages.

2. What precisely predicts emotional exhaustion of female and male physicians at different career stages? (research question 2)

As several studies have discovered, long working hours, sleep deprivation and aspects of the workplace which are at least partly influenced by colleagues and superiors, such as social support, autonomy in the job, lack of control and emotionally demanding situations are related to an increased level of stress which is associated with a higher risk of burnout [7,21,27,39-41]. In addition, it was shown that among demographic variables age and the number of children (while working more than 40 hours a week) are positively associated with exhaustion and the burnout syndrome [21,25]. Working in the surgery specialty seems to be a protective factor against burnout [42]. In this study the predictive influences of age, having children, the quality of the relationship to colleagues and support by the superior, the real working time as attendance in the clinic as well as the specialty of surgery shall be examined.

3. How do predictors of emotional exhaustion differ between female and male physicians in full-time leading position (senior physicians) with the possibility of having children? (research question 3)

Since there exist no studies on the special stressors among senior physicians with children and, moreover, female physicians in this group are a relatively new but prospectively growing phenomenon, this question remains an open one.

## Methods

### Description of study participants

The present cross-sectional study is based on data from a survey among German hospital physicians in 2007. At that time, all hospital physicians were listed in the Medical Register of the city of Hamburg, Germany. Eligible physicians, who were resident in Germany, not retired and, therefore, not over 64 years old received an 8-page questionnaire as well as an accompanying letter describing the goals of the study, assuring anonymity and asking for a reply within 14 days via a reply paid envelope. From 4,399 listed hospital physicians, 4,280 physicians fulfilled the criteria. Every second was contacted. Nine hundred and ninety-four physicians completed the questionnaire, providing a return rate of 46.5%. Participants were not compensated for study participation. The study was approved by the ZfAM’s institutional review board according to the guidelines of the National Institutes of Health, USA, and the Helsinki Declaration.

### Measures

The participants received a 60-item questionnaire covering e.g. these issues.

- *Demographics* included age, gender, number of children and marital status.
- *General characteristics of the work place and contract* included, for example, the career level as a junior or senior physician, with the latter including section heads. A later group variable combined the position with sex (group 1: junior physician/male, 2: junior physician/female, 3: senior physician/male, 4: senior physician/female). Other issues were part-time or full-time contract and discipline.
- Questions relating the *current work situation* concerned aspects like the time of clinical attendance (“How many hours did you spend at the clinic last week (including on-call-duties, lunch breaks etc.)?”), the number of all duties in the preceding month, the quality of the relationship to colleagues (“How is your relationship to your colleagues?”, Likert item: very good, 1 → very bad, 5) and the quality of support by the superior (“How is the support by your superior?”, Likert item: very good, 1→ very bad, 5).
- Among *psychomental health aspects* the risk of burnout was assessed on a German version of the 22-item version of the Maslach Burnout Inventory (MBI). This translation of the original MBI proved to be a stable measure instrument across different professional groups [43]. The MBI [44-46] assesses three dimensions of the risk of developing burnout syndrome using 7-stage frequency scales (never to daily), with emotional exhaustion (EE), depersonalization and personal accomplishment. Analyses focused on the MBI-scale emotional exhaustion (assessed by nine items, e.g. “I feel emotionally drained from my work”) which is considered as the best predictor of the risk of developing burnout syndrome [21-24,30,31]. According to Maslach and Jackson [46] the risk of burnout was rated as “high” when the value on the MBI-scale emotional exhaustion corresponded to 26 points or more.

### Statistical analyses

All analyses were performed with PASW Statistics, SPSS Version 18.0. First, descriptive statistics are reported for the four groups of junior and senior physicians with and without children. Only physicians with full-time weekly work commitments and without interruptions of work caused by holidays or illness during the last week (N = 752) were included into the analyses according research question 1 until 3. This constriction was necessary since the part-time working collective was too small for analyses between junior and senior physicians. Subsequent group comparisons according to research question 1 and focusing on child-rearing physicians were tested by an univariate covariance analysis (ANCOVA). Prior to that, the relevant covariates of clinical attendance during the last week and recovery time during clinical attendance last week were determined through correlation analyses. Results were supported by χ^2^-tests and t-tests. Based on results obtained by descriptive analyses, four listwise multiple regressions were applied to answer research question 2 for four groups of physicians with and without children (junior/male, junior/female, senior/male, senior/female) and in the case of research question 3 we focused on the two groups of female and male senior physicians in leading positions.

## Results

Overall, the gender and age distributions are almost conform to the official total collective in the year 2010 [2,5]. With an average age of 39.9 years (SD = 9.46) the 752 physicians in the present study are sligthly younger than those in the official statitistics mentioned above. The group of participants included less women (N = 263, 35.0%) and more men (N = 489, 65.0%) than in the official statistics (43.7% women vs. 56.3% men). Participants had a working contract about 41.2 hours a week (SD = 4.0) and 11.5 years (SD = 9.2) working experience as a physician on average. The majority was married (N = 394, 52.5%) and had no children (N = 384, 51.1%).

Table 1 displays the characteristics of participants by position (junior physician, senior physician) and parenting. It is noticeable that mothers work significantly fewer hours than fathers (p = .05 for junior physicians, p = .001 for senior physicians). However, among senior physicians mothers serve the same number of duties as men (p > .05). With rising career women have less children (mean = 1.5) than men (mean = 2.4, p = .00). Support by the superior is seen rather critically with nearly the same quality (means between 2.8 and 3.0) in all groups compared. There exist no gender differences on the MBI-ratings for emotional exhaustion between junior physicians with or without children (both p > .05), whereas among senior physicians men without children report a higher self-rated burnout risk (mean = 24.6) than women (mean = 18.8, p > .02). Further, with 51% more than the double of male senior physicians without children report a high risk of burnout compared to their female colleagues (22%, p = .02). Mothers among junior physicians report a high burnout risk less often than their male colleagues (24% vs. 30%, p = .00) and far less than mothers among senior physicians do (50%). Those have a significantly higher chance to report a high burnout risk than senior male physicians with children (29%, p = .00).

**Table 1:** **Characteristics of the study participants**

	**Male**		**Female**		
**Variable according to position/** **parenting**	**N**	**Mean** **(SD)**	**N**	**Mean** **(SD)**	**Sig. p**
**Age** **(years)**					
junior, no children	151	34.8 (6.7)	190	32.3(5.2)	.000***
junior, children	135	39.0 (6.9)	38	42.9 (9.3)	.02*
senior, no children	38	44.8 (6.8)	36	45.0 (8.0)	
senior, children	176	48.6 (7.6)	23	46.9 (7.7)	
**Work experience** **(years)**					
junior, no children	154	6.2 (5.9)	193	4.8 (4.6)	.02*
junior, children	137	9.8 (6.5)	38	11.5 (8.9)	
senior, no children	40	15.8 (6.4)	36	17.5 (8.2)	
senior, children	178	20.8 (7.9)	23	18.5 (7.0)	
**Clinical attendance** **(hours last week)**					
junior, no children	152	55.3 (12.2)	192	55.3 (12.2)	
junior, children	132	56.4 (11.0)	37	52.4 (9.4)	.05*
senior, no children	39	56.1 (13.6)	36	54.9 (14.1)	
senior, children	178	57.8 (10.1)	23	50.1 (11.7)	.001**
**Number of duties** **(last month)**					
junior, no children	152	4.9 (3.1)	191	5.0 (3.1)	
junior, children	136	6.1 (3.5)	38	5.0 (2.8)	.04*
senior, no children	38	7.0 (3.8)	36	6.8 (4.0)	
senior, children	174	7.7 (4.8)	20	7.3 (4.5)	
**Number of children**					
junior, children	136	1.8 (0.8)	38	1.8 (0.7)	
senior, children	176	2.4 (1.0)	21	1.5 (0.8)	.000***
**Relationship to colleagues**					
junior, no children	154	1.7 (0.7)	192	1.7 (0.7)	
junior, children	137	1.8 (0.6)	38	1.8 (0.6)	
senior, no children	40	1.8 (0.8)	36	1.9 (0.7)	
senior, children	179	1.8 (0.7)	23	1.9 (0.6)	
**Support by superior**					
junior, no children	153	3.0 (1.1)	192	3.0 (1.1)	
junior, children	137	2.9 (1.1)	38	3.0 (1.2)	
senior, no children	40	2.8 (1.2)	36	2.3 (1.0)	
senior, children	169	2.8 (1.1)	23	2.9 (1.3)	
**Emotional exhaustion** **(MBI)**					
junior, no children	152	22.7 (11.0)	192	22.7 (9.6)	
junior, children	135	21.7 (11.0)	37	20.4 (10.1)	
senior, no children	39	24.6 (11.6)	36	18.8 (9.5)	.02*
senior, children	174	20.2 (11.5)	22	24.8 (9.9)	
	**N**	**%**	**N**	**%**	
**High risk of burnout**					
junior, no children	52	0.34	66	0.34	
junior, children	41	0.30	9	0.24	.000***
senior, no children	20	0.51	8	0.22	.02*
senior, children	51	0.29	11	0.50	.000***

Criteria: only physicians with full-time contract, no other restrictions; relationship to colleagues (very good, 1 → very bad, 5); support by superior (very good , 1 → very bad, 5); high risk of burnout = EE > 26; *p ≤ .05, **p ≤ .01, ***p ≤ .001 (pairwise χ2-test/t-test).

### Differences in emotional exhaustion (research question 1)

For the overall comparison of the dependent variable emotional exhaustion between the four groups of physicians with children (group 1: junior physician/male, 2: junior physician/female, 3: senior physician/male, 4: senior physician/female) an univariate covariance analysis (covariates: clinical attendance, recovery time) was performed. The result did not confirm hypothesis 1 (F = .91, *df* = 3, p = .44, η^2^ = .008), that parenting physicians at different career stages do differ significantly on the variable emotional exhaustion.

### Predictors of emotional exhaustion (research question 2)

The results of four listwise multiple regression analyses (regression 1: junior male physicians, regression 2: junior female physicians, regression 3: senior male physicians, regression 4: senior female physicians) are reported.

All regression models included the same independent factors that were set up in correlation analyses (age, surgery specialty, children, relationship to colleagues, support by superior, clinical attendance). On this basis the model for junior male physicians (regression 1) explained 13% (adjusted R^2^) of the variance of emotional exhaustion, in the second regression model with junior female physicians 16% of the variance of emotional exhaustion were explained. Equivalent, for senior male physicians (regression 3) 27% of the variance of emotional exhaustion were explained and for senior female physicians (regression 4) 39% of the variance. Table 2 summarizes the results.

**Table 2:** **Predictors of emotional exhaustion** (**multiple regression analyses**)

	**Junior** **/male** **(regression 1)**				**Junior** **/female** ** (regression 2)**				**Senior** **/male** **(regression 3)**				**Senior** **/female** **(regression 4**)			
**Predictor**	**std. beta**	**SE beta**	**t**	**p**	**std. beta**	**SE beta**	**t**	**p**	**std. beta**	**SE beta**	**t**	**p**	**std. beta**	**SE beta**	**t**	**p**
children	-0.08	1.32	-1.25	.21	-0.12	2.05	-1.52	.13	-0.09	1.91	-1.44	.152	0.26	2.27	2.25	.03*
relationship to colleagues	0.20	1.00	3.20	.00**	0.03	0.92	0.40	.69	0.22	1.11	3.39	.001**	0.41	1.75	3.45	.00***
support by superior	0.27	0.59	4.28	.00***	0.37	0.59	5.34	.00***	0.41	0.71	6.32	.00***	0.31	1.01	2.62	.01**
age	0.01	0.09	0.21	.84	0.08	0.11	0.99	.32	-0.04	0.10	-0.60	.551	-0.27	0.14	-2.26	.03*
clinical attendance	0.05	0.06	0.82	.42	0.09	0.06	1.40	.16	0.03	0.08	0.44	.66	0.42	0.10	3.56	.00***
surgery specialty	-0.01	1.27	-0.23	.817	-0.11	1.32	-1.59	.113	-0.07	1.49	-1.06	.291	-0.15	2.29	-1.21	.23
**adj. R**-**Square**	0.13				0.16				0.27				0.39			
**N**	259				202				193				52			

Criteria: only physicians with full-time weekly work commitments, without interruptions of work caused by holidays or illness during the last week; Dependent variable: emotional exhaustion (MBI). Children (no/yes), relationship to colleagues (very good → very bad), support by superior (very good → very bad), age (years), clinical attendance (hours last week), surgery specialty (no/yes), *p ≤ .05. **p ≤ .01. ***p ≤ .001.

As the results show, support by the superior is the only variable which significantly predicts emotional exhaustion in all groups. The more critical the level of support is rated, the more probable higher levels of emotional exhaustion are. Except for the group of female senior physicians, the experienced support by the superior is the most important predictor of emotional exhaustion. The quality of the relationship to colleagues is another crucial predictor in nearly all groups, except female junior physicians. For men, the experienced support by the superior (β_junior/male_ = .27, p = .00; β_senior/male_ = .41, p = .00) is more important than the quality of the relationship to colleagues (β_junior/male_ = .20, p = .00; β_senior/male_ = .22, p = .00), regardless of the career stage they are in. For women predictors of emotional exhaustion differ between junior and senior physicians. For female junior physicians the only influencing factors is the support by the superior (β_junior/female_ = .37, p = .00). With increasing responsibility more predictors of emotional exhaustion become relevant which will be illustrated in the following paragraph.

### Predictors of emotional exhaustion in leading positions (research question 3)

The last two columns of Table 2 show the results on emotional exhaustion of female and male physicians in full-time management position (senior physicians). For men, the experienced support by the superior is the most important factor (β = .41, p = .00), followed by the quality of the relationship to colleagues (β = .22, p = .00). Furthermore, additive factors play a role for emotional exhaustion among female senior physicians. Here the clinical attendance during the last week has the most impact on emotional exhaustion (β = .42, p = .00) meaning that the longer the overall working time was, the higher emotional exhaustion is rated. The more critical the relationship to colleagues (β = .41, p = .00) and support by the superior (β = .31, p = .01) are seen, the higher emotional exhaustion is. Not least, for women age (β = -.27, p = .03) is a negative predictor of emotional exhaustion while having children (β = .26, p = .03) correlates positively, respectively critically, with it. Thus, having children becomes a significant predictor of burnout only in the group of female senior physicians.

## Discussion

With the prospect of an increasing number of female physicians in leading positions in the next years, the present study aimed to highlight especially the psychological strain for mothers among physicians right now. The situation of female hospital physicians in leading positions is characterized by multiple roles which all together require a special balancing act between the profession as a physician, leadership responsibility and family tasks. Conflicts between work and family seem particularly probable at this career level. Until now, the combination of being a mother and a full-time leader in the medical field while highlighting the risk of burnout was no research topic yet. Former studies showed that maternity itself seems to be a protective factor against burnout [26].

The current findings limit the protective effect of motherhood for the psychological well-being in some circumstances. If medical executives regard themselves as emotional exhausted, depends on different conditions for women and men. For men, equal to their career level, good relations to colleagues and support by their superior are of first priority as not to burn out (research question 2). Support by the superior is also important for women at the beginning of their career, however, with rising responsibility emotional exhaustion additionally depends on their clinical attendance, relationship to colleagues, age, and, moreover, on the question whether they are mothers or not (research question 3). Interestingly, it seems as if there is a leap in severity of emotional exhaustion among mothers with growing responsibility, respectively, with the switch from the position as a junior physician to senior physician (from 24% to 50%) which cannot be confirmed for men (research question 1). Although the covariate analysis (research question 1) does not confirm differences in the level of emotional exhaustion due to statistical limitations, the accompanying t-tests provide evidence. This is a strong indication for an incremental stress situation in female physicians with increasing management tasks who work (50 hours on average) even more than female managers in other professional domains [36]. Even though mothers do not attend the clinic as long as their male colleagues, they serve the same amount of duties.

Male senior physicians with children seem to be able to focus more on their job which is reflected in their significantly longer clinical attendance compared to their female counterparts. This might be explained by the circumstance that their spouses unburden them to a high degree in family matters. According to this, Hohner et al. [34] reported that 74% of Germany’s male physicians have spouses who do not work at all or who have part-time jobs. By contrast, female physicians in top positions often have spouses just as successful as themselves and therefore women might tend to engage stronger in family life. Thus, the integration of family and job becomes an own objective for women in leading positions [10]. So far, the lack of suitable support measures from employers is an additional strain. Furthermore, also male executives without children report frequently high emotional exhaustion, comparable to the level of senior physician mothers. This fits to Ahola’s finding that unmarried and thus rather childless men have a higher risk of burnout [47].

Different limitations of the study should be considered. The present study follows a cross-sectional design without control group. Due to the design, possible confounders could not be controlled for and causal conclusions cannot be drawn. Future longitudinal multimethod research designs could gather detailed information and causal attributive conclusions, e.g. on the influential factors on burnout.

Some groups, e.g. female senior physicians, include only few cases. This reflects that, women, and additionally those with children, in fact are a relatively small group in hospitals these days. It is possible that this effects test statistics and might explain the non-significant result in the first research question. We cannot exclude selection effects, for instance, by voluntary study participation. It might be possible that women belonging to the relatively small group of female senior physicians in Hamburg have had refused to participate because of the fear of losing anonymity through the potential combination of demographic and workplace details.

## Conclusions

The protective effect of having children on the feeling of emotional exhaustion is not emphasized for all physicians by our findings. Aspects of the work situation like support by superiors as well as relationship to colleagues seem to be more important for physicians’ psychological well-being at different career levels, regardless, if they are parents or not. Parenthood just becomes a crucial but critical predictor of emotional exhaustion with the combination of parenting and working full-time on a leading position as a woman. Hence, the group of female senior physicians seems to be characterized by a triple burden. That, all in all, a compensation of psychological strain is possible, can be seen in the group of senior physician fathers. Modifications of law alone, as it was indicated by the Working Time Act in 2004, do not seem to be sufficient to build a bridge between work and family, especially for employed female hospital physicians in Germany. Innovative approaches by employing hospitals become more urgent. Still, nurseries and company kindergartens with flexible and extended childcare hours including weekend shifts and duties, are scarce and do not cover the current need of employed physicians yet. As a consequence, with new approaches in supportive childcare and worktime models German hospitals could strengthen the reconcilability of family and working life besides duties and leadership for those female physicians with children already working in leading positions but especially for those who are interested in a future medical career.
